# Editorial: A new perspective on the importance of dendritic cells in cancer immunity: from bench to patient’s bedside

**DOI:** 10.3389/fimmu.2026.1860443

**Published:** 2026-05-01

**Authors:** Domenico Galati, Zohreh Amoozgar, Ling Ni

**Affiliations:** 1 Hematology-Oncology and Stem Cell Transplantation Unit, Istituto Nazionale Tumori-IRCCS-Fondazione 'G. Pascale, Napoli, Italy; 2 Massachusetts General Hospital, Harvard Medical School, Boston, MA, United States; 3 Institute for Immunology and School of Basic Medicine, Tsinghua University, Beijing, China

**Keywords:** cancer immunity, DC vaccines, DC-based cell therapy, dendritic cells, immunotherapy

Dendritic cells (DCs) are widely recognized as central orchestrators of antitumor immunity and remain an important focus in the development of cancer immunotherapy. As professional antigen-presenting cells, they integrate antigen sensing, processing, presentation, co-stimulatory signals, and microenvironmental cues to shape the quality, magnitude, and durability of T-cell responses. This biological role has long supported the idea that DCs could be exploited therapeutically. However, clinical experience has revealed that the impact of DC-based strategies is highly variable across tumor types and treatment settings. A major reason is that tumor-infiltrating DCs are not simply defective effectors. Rather, they behave as dynamic and context-dependent regulators whose differentiation, activation state, and function can be profoundly altered by the tumor microenvironment (TME) (1, 2).

This Research Topic, *“A New Perspective on the Importance of Dendritic Cells in Cancer Immunity: From Bench to Patient’s Bedside,”* was conceived to address this complexity. Its goal was to examine how a more refined understanding of DC biology may support the development of more effective and personalized immunotherapeutic approaches. Particular emphasis is placed on mechanisms that regulate DC function in cancer, the ways in which tumors subvert these cells, and the strategies that may restore or exploit their immunostimulatory potential. Overall, the contributions gathered here reflect a field that is moving beyond a simplified view of DC deficiency toward a more nuanced appreciation of DC plasticity, heterogeneity, and therapeutic tractability.

A central message emerging from this Research Topic is that the relevance of DC-associated pathways in cancer is highly context-dependent. This point is illustrated by Galán et al., whose study shows that SHP-1 depletion in CD11c^+^ cells impairs, rather than enhances, antitumor immunity. Additional analyses in XCR1^+^ and Lyz2^+^ compartments further support contributions from both cDC1s and macrophages. These findings challenge the assumption that manipulation of intracellular negative regulators will uniformly potentiate antitumor responses. Instead, they emphasize that the outcome of pathway modulation depends on both the cellular compartment targeted and the TME in which these cells operate (1, 2).

The Topic also highlights that therapeutic progress will depend not only on mobilizing DCs, but also on understanding how different DC populations are generated and functionally programmed. In acute myeloid leukemia, Rejeski et al. show that the *ex vivo* generation of leukemia-derived DCs, the induction of immune activation, and leukemic blast lysis are strongly influenced by the concentration of immunomodulatory agents used during preparation. In a complementary direction, Zheng et al. report that FLT3L/GM-CSF-induced DCs are enriched in conventional DC subsets and exhibit enhanced cross-presentation, broader tumor-specific CD8^+^ T-cell responses, and greater capacity to remodel the TME than conventional GM-CSF/IL-4-generated DCs. Together, these studies underscore that DC-based immunotherapy cannot be reduced to antigen loading alone, but must also account for the developmental route, subset composition, and functional programming of the DC product itself (3).

Another important message arising from this Research Topic is that DC dysfunction varies substantially across malignancies. As discussed by Jordan et al. multiple myeloma provides a representative example of disease-specific immune disruption in which malignant plasma cells profoundly alter DC abundance, phenotype, and function. The defects in antigen presentation and T-cell priming contribute to immune escape and persistent disease. This perspective reinforces the need to align therapeutic design with the biology of each disease setting rather than applying uniform strategies across biologically distinct cancers (2).

Importantly, this Research Topic broadens the translational horizon of the field beyond classical cell-based vaccination. Redkin and Turubanova examine DC-derived exosomes as cell-free anticancer agents and discuss approaches to enhance their immunogenicity. Their review highlights the possibility that key immunostimulatory functions of DCs may be harnessed in more flexible and engineerable formats, thereby extending the conceptual and therapeutic scope of DC-based intervention (3).

Taken together, the articles in this Topic place DC biology in a broader and more clinically relevant framework. They show that DCs in cancer are shaped by tumor-driven suppression, differ across disease contexts, and remain amenable to therapeutic reprogramming. This integrated view may also help explain why the compelling rationale for DC-based immunotherapy has not yet translated into uniform clinical benefit, while still recognizing meaningful progress in selected settings such as sipuleucel-T in metastatic castration-resistant prostate cancer and autologous tumor lysate-loaded DC vaccination in glioblastoma (4, 5). More broadly, the Research Topic suggests that future advances will depend on better patient stratification, improved control of tumor-induced immune suppression, closer alignment between therapeutic platforms and DC subset biology, and more effective integration with complementary treatment modalities.

Overall, this Research Topic frames DCs not as a single therapeutic entity, but as a diverse and adaptable immunological system whose functions can be disrupted, redirected, and potentially exploited with greater precision than previously appreciated. In this regard, the studies presented here not only summarize current knowledge but also define the conceptual and translational priorities likely to shape the next phase of DC-based cancer immunotherapy.
